# HIF-1α Stabilization in Flagellin-Stimulated Human Bronchial Cells Impairs Barrier Function

**DOI:** 10.3390/cells11030391

**Published:** 2022-01-24

**Authors:** Ivan Ramirez-Moral, Bianca L. Ferreira, Joe M. Butler, Michel van Weeghel, Natasja A. Otto, Alex F. de Vos, Xiao Yu, Menno D. de Jong, Riekelt H. Houtkooper, Tom van der Poll

**Affiliations:** 1 Center of Experimental and Molecular Medicine, Amsterdam Infection & Immunity Institute, Amsterdam UMC, University of Amsterdam, 1105 AZ Amsterdam, The Netherlands; bianca.rodrigueslima@gmail.com (B.L.F.); j.m.butler@amsterdamumc.nl (J.M.B.); n.a.otto@amsterdamumc.nl (N.A.O.); a.f.devos@amsterdamumc.nl (A.F.d.V.); t.vanderpoll@amsterdamumc.nl (T.v.d.P.); 2 Division of Infectious Diseases, Department of Medicine, Escola Paulista de Medicina, Universidade Federal de Sao Paulo, Sao Paulo 04023-062, Brazil; 3 Laboratory Genetic Metabolic Diseases, Amsterdam Gastroenterology, Endocrinology and Metabolism, Amsterdam Cardiovascular Sciences, Amsterdam UMC, University of Amsterdam, 1105 AZ Amsterdam, The Netherlands; m.vanweeghel@amsterdamumc.nl (M.v.W.); r.h.houtkooper@amsterdamumc.nl (R.H.H.); 4 Core Facility Metabolomics, Amsterdam UMC, University of Amsterdam, 1105 AZ Amsterdam, The Netherlands; 5 Department of Medical Microbiology, Amsterdam UMC, University of Amsterdam, 1105 AZ Amsterdam, The Netherlands; x.yu@amsterdamumc.nl (X.Y.); m.d.dejong@amsterdamumc.nl (M.D.d.J.); 6 Division of Infectious Diseases, Amsterdam UMC, University of Amsterdam, 1105 AZ Amsterdam, The Netherlands

**Keywords:** immunometabolism, HIF1α, airway epithelial cells, flagellin, inflammation

## Abstract

The respiratory epithelium provides a first line of defense against pathogens. Hypoxia-inducible factor (HIF)1α is a transcription factor which is stabilized in hypoxic conditions through the inhibition of prolyl-hydroxylase (PHD)2, the enzyme that marks HIF1α for degradation. Here, we studied the impact of HIF1α stabilization on the response of primary human bronchial epithelial (HBE) cells to the bacterial component, flagellin. The treatment of flagellin-stimulated HBE cells with the PHD2 inhibitor IOX2 resulted in strongly increased HIF1α expression. IOX2 enhanced the flagellin-induced expression of the genes encoding the enzymes involved in glycolysis, which was associated with the intracellular accumulation of pyruvate. An untargeted pathway analysis of RNA sequencing data demonstrated the strong inhibitory effects of IOX2 toward key innate immune pathways related to cytokine and mitogen-activated kinase signaling cascades in flagellin-stimulated HBE cells. Likewise, the cell–cell junction organization pathway was amongst the top pathways downregulated by IOX2 in flagellin-stimulated HBE cells, which included the genes encoding claudins and cadherins. This IOX2 effect was corroborated by an impaired barrier function, as measured by dextran permeability. These results provide a first insight into the effects associated with HIF1α stabilization in the respiratory epithelium, suggesting that HIF1α impacts properties that are key to maintaining homeostasis upon stimulation with a relevant bacterial agonist.

## 1. Introduction

Under normal conditions, the human airways are protected from potentially harmful bacteria from the environment by a complex interplay between the respiratory epithelium and tissue-resident immune cells [1]. However, chronic respiratory disorders, such as chronic obstructive pulmonary disease (COPD) and cystic fibrosis (CF), render patients vulnerable to infection. One common feature of chronic respiratory diseases is areas of hypoxia in the airways [2,3]. Although hypoxia has been associated with the recurrence of infections [4], the effect of hypoxia in the airway epithelium and the mechanisms by which these cells contribute to the enhanced susceptibility to infections remain poorly understood.

Composed of mucus-secreting and ciliated cells on the most apical side, and secretory cells able to produce antimicrobial mediators intercalated, the human respiratory epithelium acts as a physical barrier preventing bacterial translocation and dissemination in the host [5]. Additionally, the respiratory epithelium is equipped with a repertoire of pattern-recognition receptors (PRRs), which sense conserved microbial components; among these, Toll-like receptor 5 (TLR5) is of great importance in the epithelium [6]. Flagellin is the unique natural TLR5 agonist, and it is expressed by several mucosal pathogens, including *Pseudomonas (P.) aeruginosa*. This Gram-negative bacterium is often found colonizing CF airways [2], and it is a common causative pathogen in infections in COPD patients [7].

Immune cells undergo major metabolic changes upon exposure to a variety of stimuli, including bacteria and components thereof [8]. In this context, many cells rapidly activate glycolysis upon stimulation in order to fulfill the immediate energy demands of an inflammatory response. Hypoxia inducible factor-1 (HIF1) has been implicated as a master regulator of metabolism in different cell subsets [8,9]. HIF1 is composed of two subunits, of which HIF1β is constitutively expressed and HIF1α is expressed in an oxygen dependent way. In the presence of oxygen, HIF1α is hydroxylated by prolyl-hydroxylase (PHD)2 [9] and then recognized by the von Hippel–Lindau (VHL) protein, leading to the proteasomal degradation of HIF1α [9]. However, in hypoxic conditions, the activity of PHD2 is reduced, resulting in HIF1α stabilization, translocation to the nucleus and dimerization with HIF1β, thereby initiating a wide range of functions [9]. HIF1α is a key regulator of glycolysis by virtue of its capacity to induce the transcription of the genes encoding proteins that enhance glucose transport and glycolysis [8,9]. In addition, HIF1 activation leads to the regulation of key transcription factors involved in cellular responses, including inflammatory processes and cell survival [4].

We recently demonstrated that, besides different leukocyte subsets, primary human bronchial epithelial (HBE) cells also utilize glycolysis to produce immune mediators after stimulation with flagellin [10]. Here, we aimed to investigate the impact of HIF1α stabilization on the metabolism and function of primary HBE cells in response to flagellin. Studies on the interaction between flagellin and airway epithelial cells are of relevance not only in the context of infections caused by bacteria that express flagellin, but also because the local administration of purified flagellin is under investigation as a potential mucosal immune adjuvant in the treatment of respiratory tract infections [11].

## 2. Materials and Methods

### 2.1. HBE Cell Differentiation

HBE cells were obtained anonymously from 3 donors undergoing a lobectomy for lung cancer at the Amsterdam University Medical Centers (A-UMC), the Netherlands. A pathologist resected the healthy tracheobronchial tissue distant from tumorous tissue and assessed the absence of malignant cells via microscopy. HBE cells were isolated according to Fulcher’s protocol [12], as described before [10]. Briefly, passage 2 (P2) to P4 cells were used for differentiation in 24-well Transwell inserts (Corning, Corning, NY, USA) treated with human type IV placental collagen in submerged PneumaCult-Ex Plus media (StemCell Technologies, Vancouver, BC, Canada). When confluent, the media was replaced by PneumaCult-ALI medium (StemCell Technologies) on the basolateral side and the media on the apical side was removed, forming an air–liquid interface. The basolateral media were renewed every two or three days for around 30 days until cells reached full differentiation.

### 2.2. HBE Cell Treatment and Stimulation

HBE cells were kept in a PneumaCult-ALI medium containing penicillin and streptomycin and stimulated with 1 μg/mL flagellin from *Pseudomonas aeruginosa* (Invivogen, Toulouse, France) or PBS (control) added to the apical compartment. Unless stated otherwise, HBE cells were stimulated for 24 h. In some experiments, HBE cells were pre-incubated for 1 h before adding the stimuli with the PHD2 inhibitor IOX2 (50 μM; MedChem Express, Monmouth Junction, NJ, USA) or vehicle (PBS + DMSO 0.05%). Cell culture supernatants and apical washes were stored at −80 °C before being analyzed. Cells were lysed in Lysis/Binding Buffer (Roche, Basel, Switzerland) and stored at −80 °C for RNA isolation or directly stored at −80 °C for metabolomics or at −20 °C for Western blot, as detailed below.

### 2.3. Metabolomics

Metabolomics was performed as described previously [10,13]. Briefly, the polar fraction was extracted through the addition of chloroform/methanol/water (2/1/1) containing the internal standards to the dry cell pellet. Then, the sample was dried and reconstituted in 100 µL methanol/water (6/4; *v*/*v*) before being applied to the ultra-high-pressure liquid chromatography system (Thermo Fisher Scientific, Waltham, MA, USA) coupled to a Thermo Q Exactive (Plus) Orbitrap mass spectrometer (Thermo Fisher Scientific). Data were analyzed using Xcalibur software (version 3.0, Thermo Fisher Scientific). Metabolite abundance was normalized to internal standards. For metabolite identification, a combination of accurate mass, (relative) retention times and fragmentation spectra, compared to the analysis of a library of standards, were used.

### 2.4. RNA Isolation and RT-PCR

Total RNA was isolated using the High Pure RNA Isolation Kit (Roche) according to the manufacturer’s instructions. cDNA was synthetized using the M-MLV Reverse Transcriptase kit (Promega, Madison, WI, USA) in the presence of an RNase inhibitor (Thermo Fisher Scientific) with 300 ng of DNase I (Roche) treated total RNA. The RT-PCR was performed on a LightCycler 480 (Roche) using the SensiFAST SYBR No-ROX Kit (Bioline, London, UK). Data are normalized to the housekeeping gene *HPRT*. The following primers were used: *HIF1A* Fwd: GCTGAAGACACAGAAGCAAAGAACCCA; Rev: CGCTTTCAGGGCTTGCGGAACT and *HPRT* Fwd: GGATTTGAAATTCCAGACAAGTTT; Rev: GCGATGTCAATAGGACTCCAG.

### 2.5. Immunoblot Analysis

Cells were lysed in RIPA buffer supplemented with HALT protease and phosphatase inhibitor (Thermo Fisher Scientific) and stored at −20 °C until processing. For HIF1α blots, samples were heated for 5 min at 95 °C. Samples were loaded in 10% polyacrylamide precast gels (Bio-Rad, Hercules, CA, USA) and transferred to PVDF membranes. After blocking for 1 h at room temperature, membranes were incubated with a rabbit anti-HIF1α antibody (14179; Cell Signaling Technology, Danvers, MA, USA), rabbit anti-β-Actin (4967L; Cell Signaling). A goat anti-rabbit (7074S; Cell Signaling) HRP-linked antibody was used as a secondary antibody. Blots were incubated with the Lumi-Light detection kit (Roche) and pictures were taken using ImageQuant LAS-4000 (GE Healthcare, Chicago, IL, USA).

### 2.6. RNA Sequencing

RNA sequencing was performed in HBE cells from 3 different donors in duplicate for each condition exactly as detailed in [10]. In brief, high-quality reads were aligned against the Genome Reference Consortium Human Genome Build 38 patch release 7 (GRCh38.p7) using Bowtie2 version 2.3.4.3 [14] with default parameters. Count data were generated by means of the FeatureCounts method [15] and the differential expression was analyzed using the DESeq2 method [16] in the R statistical computing environment (R Core Team 2014. R: A language and environment for statistical computing. R Foundation for Statistical Computing, Vienna, Austria). Throughout, significance was calculated using Benjamini–Hochberg (BH) adjusted *p*-values [17].

Using the Reactome pathway knowledgebase [18], the gene set enrichment analysis (GSEA) approach [19] was applied to determine, for any given pathway, whether a pre-defined set of genes (Reactome pathways) showed statistically significant differences between two conditions.

### 2.7. Transmembrane Permeability

Transmembrane permeability was assessed by measuring it with the 4 kDa fluorescein isothiocyanate (FITC)–dextran (Sigma, St. Louis, MO, USA) flux. After stimulation, 200 μL of medium containing 2 mg/mL FITC–dextran was added to the apical side and the media in the basolateral compartments were renewed. HBE cells were incubated for 2 h at 37 °C. Fluorescence in the basolateral medium was measured using a plate reader (Biotek, Winooski, VT, USA). A standard curve of FITC–dextran in medium (8 to 0.125 μg/mL) was used to calculate the transmembrane permeability (apparent permeability coefficient or Papp), defined as the increase in the concentration of compound per time per insert area.

### 2.8. Statistics

All analyses were performed using GraphPad Prism 7.03. The number of replicates and the statistical tests used for each data set are described in the figure legends. In most cases, Student’s *t* test was used. A *p* value < 0.05 was considered statistically significant, with levels of significance indicated as follows: * *p* < 0.05; ** *p* < 0.01; *** *p* < 0.001; ns, not significant.

## 3. Results

### 3.1. IOX2 Stabilizes Flagellin-Induced HIF1α in HBE Cells

**Figure 1:**
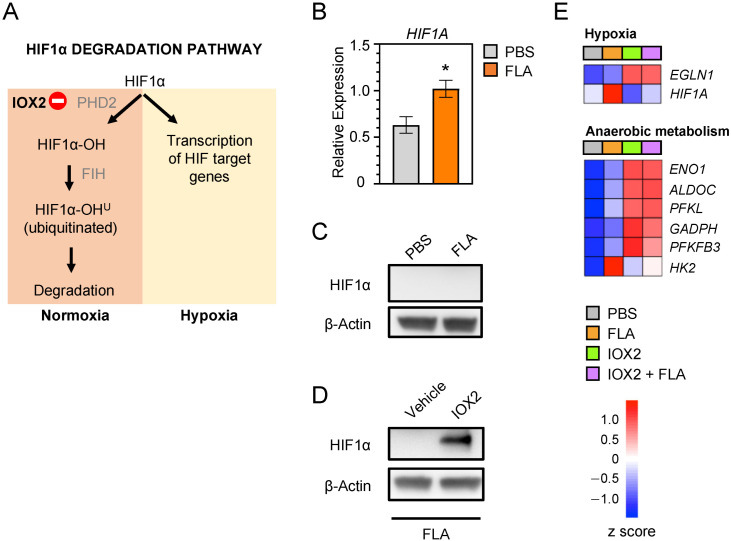
HIF1α stabilization in HBE cells (**A**) Scheme of the HIF1α degradation pathway. (**B**) Fold increase in mRNA expression for *HIF1A* analyzed via RT-PCR in HBE cells 24 h after stimulation with flagellin (1 μg/mL) or PBS (control). (**C**) HIF1α Western blot of HBE cell lysates after stimulation with flagellin or PBS for 24 h; β-actin was used as loading control. (**D**) HIF1α blot in HBE cells pre-treated for 1 h with IOX2 (50 μM) or vehicle and stimulated for 24 h with flagellin; β-actin was used as loading control. (**E**) Heat map of changes in the expression of selected genes from the HIF1α pathway in HBE cells as in (**D**). Red denotes high expression; blue indicates low expression. Data in (**B**) are presented as mean ± SEM (*n* = 6). *p* value was calculated using Student’s *t* test compared to control cells. * *p* < 0.05. Data in (**C**) and (**D**) are representative of two independent experiments wherein similar results were obtained. Data in (**E**) comprise 3 biological donors with 1–2 replicates from 2 independent experiments (*n* = 5).

The stability of HIF1α is post-transcriptionally regulated by oxygen availability through PHD2 (Figure 1A) [9]. In the presence of sufficient oxygen, PHD2 is active and hydroxylates HIF1α, thereby marking it for proteasomal degradation [9]. In hypoxic conditions, PHD2 becomes inactive, resulting in the stabilization and accumulation of HIF1α. Flagellin enhanced HIF1α mRNA (Figure 1B) but not protein expression (Figure 1C) in HBE cells. IOX2 is a potent inhibitor of PHD2 that has been shown to increase HIF1α protein levels in multiple cell types [20]. In agreement, the addition of IOX2 to HBE cells prior to stimulation with flagellin resulted in the accumulation of HIF1α (Figure 1D). RNA sequencing showed the increased expression of genes implicated in hypoxia and anaerobic metabolism in HBE cells exposed to flagellin in the presence of IOX2 when compared with cells stimulated with flagellin alone (Figure 1E).

### 3.2. IOX2 Impacts Flagellin-Induced Glycolysis in HBE Cells

**Figure 2:**
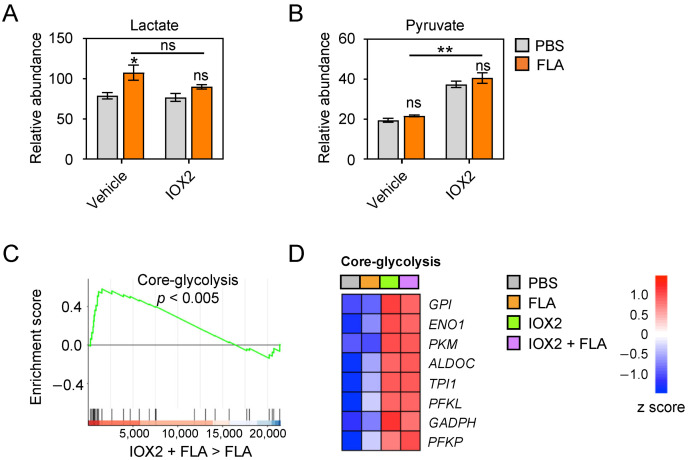
IOX2 induces glycolysis and accumulation of pyruvate in HBE cells (**A**) Relative abundance of lactate analyzed via liquid chromatography–high resolution mass spectrometry (LC-HRMS) in HBE cells pre-treated for 1 h with IOX2 (50 μM) or vehicle and stimulated for 24 h with PBS or flagellin. (**B**) Relative abundance of pyruvate as in (**A**). (**C**) Gene set enrichment analysis (GSEA) of the core glycolysis pathway in HBE cells activated with flagellin for 24 h in the presence of either vehicle or 50 μM IOX2 (pre-treated for 1 h). The x axis shows individual genes, and the y axis shows enrichment score. Red represents upregulated genes and blue represents downregulated genes. (**D**) Heat map of changes in the expression of the core glycolysis pathway genes in the indicated conditions. Data in (**A**,**B**) are presented as mean ± SEM (*n* = 3–4 replicates per group). *p* value was calculated using Student’s *t* test. * *p* < 0.05; ** *p* < 0,01; ns, not significant. Data in (**C**,**D**) comprise 3 biological donors with 1–2 replicates from 2 independent experiments (*n* = 5).

The stabilization of HIF1α through the inhibition of PHD2 has been shown to result in enhanced glycolysis in a variety of cells [4,21]. We have previously reported that flagellin induces a metabolic reprogramming of HBE cells by enhancing carbohydrate metabolism, specifically glycolysis [10]. To determine whether PHD2 inhibition further enhanced flagellin-induced glycolysis, we assessed intracellular levels of lactate. In agreement with our earlier report [10], flagellin induced the increased accumulation of lactate in HBE cells compared to the medium control (Figure 2A). In the presence of IOX2, the flagellin-induced rise in intracellular lactate levels was less clear and not statistically significant (*p* = 0.06 versus IOX2 in medium control). Remarkably, however, IOX2 caused a higher intracellular accumulation of pyruvate regardless of the presence or absence of flagellin (Figure 2B). In accordance, RNA sequencing and GSE analysis [19] indicated that IOX2 significantly impacted the flagellin-induced expression of genes encoding enzymes involved in the core glycolysis pathway (Figure 2C,D). Of interest, IOX2 strongly induced the expression of *PKM*, the gene encoding pyruvate kinase M, the rate-limiting enzyme that catalyzes the final step of glycolysis, resulting in pyruvate generation, whilst IOX2 did not affect the expression of *LDHA*, the gene encoding lactate dehydrogenase A, the enzyme responsible for the conversion of pyruvate into lactate (Figure 2D and Figure S2A). Moreover, IOX2 did not alter the expression of *PDH*, the gene encoding the enzyme pyruvate dehydrogenase, responsible for the conversion of pyruvate into acetyl-CoA (Supplementary Figure S1A). In agreement with unaltered *PDH* expression, IOX2 did not modify the activity of the TCA cycle in flagellin-stimulated HBE cells, as indicated by the GSE analysis of the TCA cycle (Supplementary Figure S1B). Collectively, these results suggest that IOX2 enhances flagellin-induced glycolysis in HBE cells, resulting in the accumulation of intracellular pyruvate.

### 3.3. IOX2 Inhibits Flagellin-Induced Transcriptional Upregulation of Cytokine Signaling Pathways

**Figure 3:**
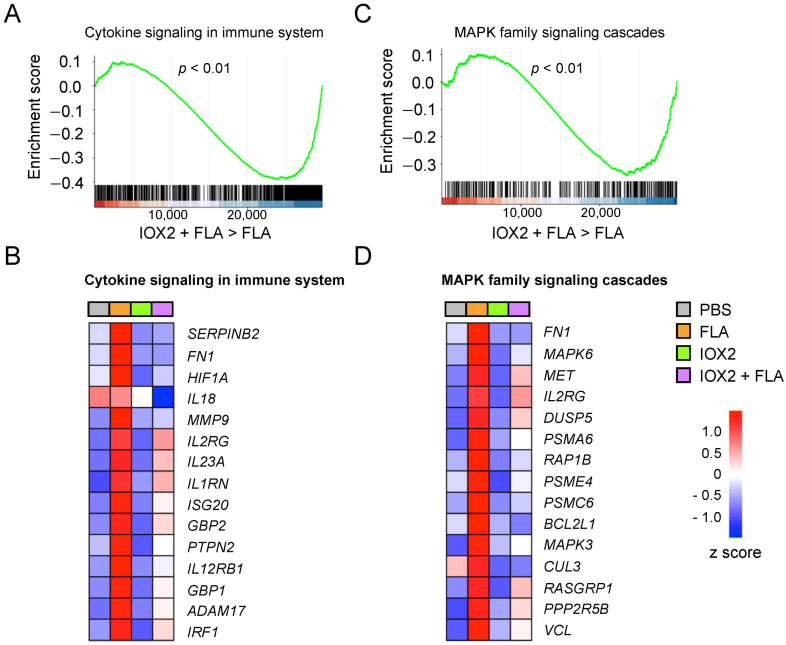
IOX2 inhibits immune signaling pathways in HBE cells. (**A**) Gene set enrichment analysis (GSEA) of the “cytokine signaling in immune system” pathway (R-HAS-1280215.5 in https://reactome.org, accessed on 8 September 2021) in HBE cells activated with flagellin or PBS for 24 h in the presence of either vehicle or 50 μM IOX2 (pre-treated for 1 h). (**B**) Heat-map of changes in the expression of the “cytokine signaling in immune system” pathway genes in the indicated conditions. (**C**) GSEA of the “MAPK family signaling cascades” pathway (R-HAS-5683057 in https://reactome.org, accessed on 8 September 2021) in HBE cells from (**A**). (**D**) Heat map of changes in the expression of the “MAPK family signaling cascades” pathway genes in HBE cells from (**A**). Data in (**A**–**D**) comprise 3 biological donors with 1–2 replicates from 2 independent experiments (*n* = 5). Red represents upregulated genes and blue represents downregulated genes. In (**A**,**C**), Log2-fold expression values were ranked in descending order. The x axis shows individual genes and the y axis shows enrichment score.

HIF1α can directly regulate the expression of a variety of genes independently of its metabolic effects [8]. An unbiased analysis of RNA sequencing data (using the Reactome Pathway Browser tool at https://reactome.org, accessed on 8 September 2021) [19], comparing the flagellin effects in HBE cells in the presence or absence of IOX2, identified several pathways significantly modified by IOX2. Untargeted pathway analysis was performed to find the top five pathways upregulated and downregulated by IOX2 in flagellin-stimulated HBE cells (Supplementary Figure S2). We recently reported a strong over-expression of signaling pathways implicated in innate immunity in HBE cells stimulated with flagellin [10]. Of interest, the top downregulated pathway in flagellin-stimulated HBE cells treated with IOX2 was “cytokine signaling in the immune system” (R-HAS-1280215.5 in Reactome) (Figure 3A and Figure S2). Among the most downregulated genes in the presence of IOX2 were interferon-induced proteins (*ISG20, GBP1, GBP2* and *PTPN2*), metalloproteins and adhesion proteins important for cell migration and host defense (*FN1, MMP9* and *ADAM17*), members of the interleukin 1 family (*IL18* and *IL1RN*) and effector cytokines and cytokine receptors (*IL2RG, IL23A* and *IL12RB1*) (Figure 3B). Additionally, “MAPK family signaling cascades” (R-HAS-5683057 in Reactome) was among the top downregulated pathways in the presence of IOX2 (Figure 3C and Figure S2), which included MAPK intermediates (*MAPK3* and *MAPK6*) and regulators (*DUSP5* and *RASGRP1*), components of the proteasomal complex (*PSMA6, PSME4* and *PSMC6*) and ubiquitination reactions (*CUL3*) (Figure 3D). Collectively, these results suggest that IOX2 inhibited the expression of many genes involved in the proinflammatory effects of flagellin in HBE cells.

### 3.4. IOX2 Prevents Flagellin-Induced Upregulation of Tight Junction Interactions

**Figure 4:**
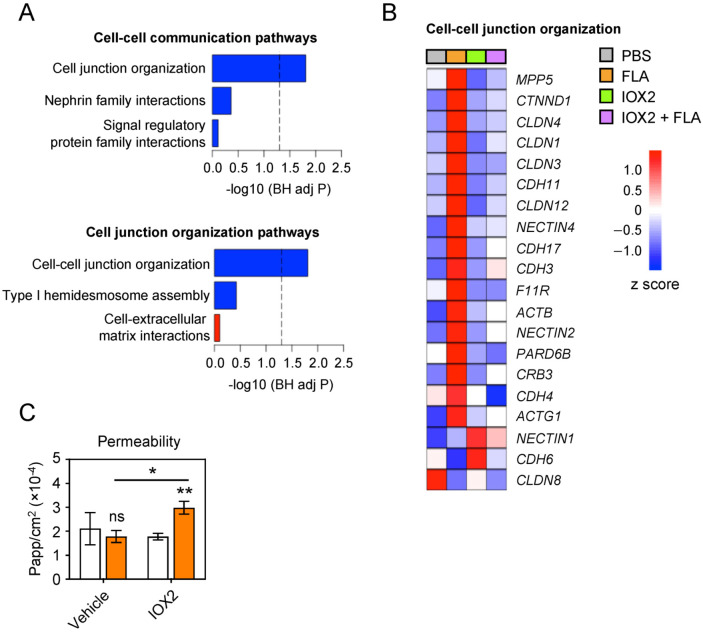
IOX2 inhibits expression of genes mediating cell–cell junction organization and compromises membrane permeability. (**A**) Cell–cell communication pathway analysis (R-HAS-1500931 in https://reactome.org, accessed on 8 September 2021) from genome-wide transcriptomic differences in HBE cells activated with flagellin for 24 h in the presence of IOX2 (added 1 h prior to stimulation) relative to that in the presence of vehicle. Data display child pathways enriched for upregulated (red) or downregulated genes (blue). (**B**) Heat map of changes in the expression of the cell–cell junction organization pathway genes in the indicated conditions. Red denotes high expression; blue indicates low expression. (**C**) Membrane integrity in HBE cells stimulated with flagellin or PBS (control) for 24 h with or without pre-incubation (1 h) with 50 μM IOX2, as measured by diffusion of FITC–dextran beads from the apical to the basolateral compartment. Data in (**A**,**B**) comprise 3 biological donors with 1–2 replicates from 2 independent experiments (*n* = 5). Data in (**C**) are displayed as mean ± SEM of 3–4 replicates representative of two experiments with similar results. *p* values were calculated using Student’s *t* test. * *p* < 0.05; ** *p* < 0.01; ns, not significant.

The maintenance of the structural integrity of the epithelial barrier is essential for homeostasis and tissue resilience, and cell–cell interactions and tight junctions are key functions herein [22]. “Cell junction organization” (R-HAS-446728.2 in Reactome) was among the top pathways downregulated by IOX2 in flagellin-stimulated HBE cells (Supplementary Figure S2). Further analysis of this “parent pathway” revealed that IOX2, especially, modified the genes implicated in tight junction interactions (Figure 4A, R-HAS-1500931 in Reactome). Genes of which the upregulation induced by flagellin was prevented by IOX2 included *CLDN1*, *CLDN3*, *CLDN4* and *CLDN12*, encoding claudins, important constituents of the tight junction complexes that mediate the permeability of epithelia [22], *CDH3*, *CDH4*, *CDH11* and *CDH17*, encoding cadherins, a family of adhesion proteins [22], and *CTNND1*, encoding a key regulator of cell–cell adhesion that associates with and regulates the cell adhesion properties of cadherins [22] (Figure 4B). Tight junctions are important for cell-to-cell adhesion in epithelial sheets, serving as a physical barrier to prevent water and solutes from passing through the paracellular space. To test the functional consequence of IOX2 in tight junctions, we determined the permeability of HBE cell cultures to dextran (Figure 4C). While flagellin did not alter permeability when compared with the medium control, IOX2 significantly increased the permeability of flagellin-stimulated HBE cell cultures without affecting the permeability of non-stimulated cultures. Together, these data suggest that flagellin enhances tight junction interactions to prevent the disruption of barrier integrity and that this protective mechanism is compromised by IOX2.

## 4. Discussion

The airway epithelium represents the first line of defense against respiratory pathogens. While it is well known that chronic respiratory disorders and hypoxia increase the risk of infections [4], studies addressing the function of the master regulator of hypoxia HIF1α in the airway epithelium are scarce, and knowledge of the consequences of HIF1α stabilization in these cells, a characteristic feature in hypoxic conditions, is limited. The present study builds upon a recent investigation from our group in which we demonstrated that the common mucosal pathogen component, flagellin, induces a glycolytic response in primary HBE cells, which was key to the induction of innate immune genes [10]. Here, we used this model system to show that HIF1α stabilization by IOX2 inhibits flagellin-induced innate immune signaling, as well as the expression of genes encoding tight junction interactions, which was accompanied by impaired epithelial barrier function. These results provide a first insight into the effects associated with HIF1α stabilization in the respiratory epithelium. We here used primary human bronchial epithelial cells. Studies of the biology of the human airway epithelium have mostly been performed using cell lines that do not recapitulate the complexity of the human tissue. Moreover, these cell lines possess intrinsic alterations; for instance, their metabolism is different when they originate from cancerous tissue [23].

HIF1α can regulate immune cell function in different ways. A well-investigated mechanism is through the control of cellular metabolism [8,9]. Specifically, HIF1α can increase the rate of glycolysis via the transcriptional enhancement of glycolytic gene expression. We recently reported a strong induction of glycolysis by flagellin in HBE cells via the activation of the mammalian target of rapamycin (mTOR) pathway [10]. We here documented that IOX2 induced a further upregulation of the genes implicated in glycolysis in HBE cells stimulated with flagellin, which was accompanied by the intracellular accumulation of pyruvate, but not lactate. Moreover, IOX2 did not impact on the activity of the TCA cycle, as reflected by the unchanged expression of genes encoding TCA enzymes. Whilst IOX2 strongly increased the expression of *PKM*, the gene encoding pyruvate kinase M (the enzyme mediating pyruvate generation), it did not affect the expression of *LDHA* or *PDH*, the genes encoding lactate dehydrogenase A and pyruvate dehydrogenase, respectively (enzymes responsible for the conversion of pyruvate into lactate or acetyl-CoA). These differential IOX2 effects on the expression of key enzymes involved in the generation and breakdown of pyruvate at least in part explains the accumulation of intracellular pyruvate in IOX2 treated HBE cells.

In general, increased intracellular glycolysis enhances the capacity of cells to mount a proinflammatory response [24,25]. Our laboratory recently reported that this association of enhanced glycolysis and innate immune signaling also can be demonstrated in primary HBE cells [10]. Thus, the stabilization of HIF1α by IOX2, resulting in increased glycolysis, might be expected to stimulate innate immune signaling. In contrast, we here documented the profound inhibitory effects of IOX2 on the expression of multiple proinflammatory genes in flagellin-stimulated HBE cells. Importantly, previous studies also reported the anti-inflammatory effects mediated by HIF1α in respiratory epithelial cells. The addition of the PHD inhibitor dimethyloxaloylglycine (DMOG) resulted in the reduced expression of the inflammatory mediators IL-6 and CXCL10 in HBE cells stimulated with either flagellin, the TLR3 agonist polyI:C or *P. aeruginosa* [26]. Similarly, the stabilization of HIF1α in primary murine alveolar epithelial cells by their exposure to hypoxia resulted in the suppression of key innate immune molecules, including granulocyte–macrophage colony-stimulating factor, CCL2 and IL-6, an effect that at least in part was reproduced by the exposure of these cells to DMOG [27]. Together, these data suggest that the anti-inflammatory and metabolic effects related to the stabilization of HIF1α in the respiratory epithelium are not mechanistically linked.

The loss of epithelial barrier function is a hallmark feature of many acute and chronic lung diseases [5]. Previous studies have indicated that hypoxia may play a role herein [4]. The exposure of A549 epithelial cells to hypoxia resulted in the stabilization of HIF1α and decreased the expression of tight junction proteins [28]. Likewise, in primary rat alveolar epithelial cells and human nasal epithelial cells, hypoxia decreased the expression of tight junction proteins and increased permeability, as measured by transepithelial electrical resistance and dextran diffusion [28,29]. However, other investigations have suggested hypoxia and/or HIF stabilization may exert barrier protective effects. For example, in immortalized human bronchial epithelial cells and mouse primary tracheal epithelial cells, hypoxia, or treatment with DMOG, protected against the loss of epithelial barrier function during the oxidative stress elicited by exposure to hydrogen peroxide [30], and in a mouse model of Fas ligand-induced lung injury, DMOG attenuated the increase in alveolar permeability, as measured by IgM levels in the bronchoalveolar lavage fluid [31]. In addition, the inhibition of PHDs in the intestinal epithelium has also been found to preserve, rather than disrupt, barrier function [32]. We here showed that flagellin induced a strong upregulation of the genes encoding claudins and cadherins, and that IOX2 prevented these flagellin-induced changes and, accordingly, impaired epithelial barrier function, as measured by dextran permeability. 

Our study has limitations. The intracellular levels of pyruvate, as shown in Figure 2B, are influenced by a concerted action of synthesis (through glycolysis) and consumption (through lactate formation, entry in mitochondria, etc.), as well as export from the cell through the monocarboxylate-1 transporter; based on the steady-state metabolomics analysis performed here, the equally increased pyruvate levels observed in the presence of IOX2, regardless of flagellin stimulation, cannot be readily explained. Finally, RNA sequencing data were not validated by the RT-PCR.

Hypoxia is a common phenomenon in inflammatory conditions in general, and chronic respiratory disorders in particular. The stabilization of HIF1α, the key regulator in hypoxic conditions, strongly impacted the responses of primary HBE cells to flagellin, a relevant bacterial agonist, characterized by reduced innate immune signaling and impaired barrier function. These results suggest that HIF1α affects the properties of the respiratory epithelium that are key to maintaining homeostasis upon stimulation with a relevant bacterial agonist.

## Data Availability

The datasets generated for this study can be found in GEO under the accession number GSE164704.

## Supplementary Materials

The following supporting information can be downloaded at: https://www.mdpi.com/article/10.3390/cells11030391/s1, Figure S1: Glycolysis and TCA cycle pathway analysis, Figure S2: Genome-wide transcriptome RNAseq analysis of flagellin-stimulated HBE cells in the presence of IOX2.
