# Supplementary material for: HIF-1α Stabilization in Flagellin-Stimulated Human Bronchial Cells Impairs Barrier Function
Source: Cells. 2022 Jan 24;11(3):391. doi: 10.3390/cells11030391 (PMC8834373; doi:10.3390/cells11030391)
Supplement: Supplementary file 1 [file cells-11-00391-s001.zip › cells-1544282-supplementary.pdf]

# Supplementary Material

A

B

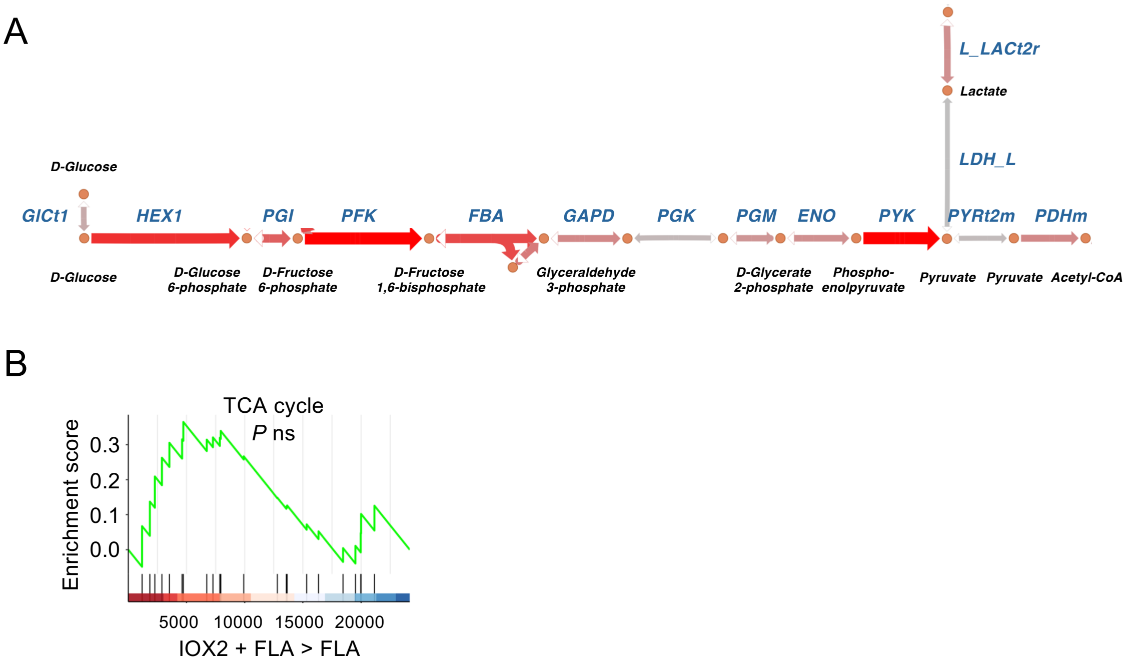


**Figure S1. Glycolysis and TCA cycle pathway analysis** (A) Schematic representation of gene expression in the glycolysis pathway in HBE cells stimulated with flagellin for 24 hours in the presence of IOX2 relative to vehicle. Each arrow represents an enzymatic reaction catalyzed by an enzyme (encoded by known genes) between two metabolites depicted by dots. Red denotes over-expression and blue under-expression of the genes involved in those reactions. A darker color indicates a bigger change in transcript expression. (B) GSEA of the TCA cycle in HBE cells from (A). Log2-fold expression values were ranked in descending order. The x axis shows individual genes and the y axis shows enrichment score. Red represents up-regulated genes and blue represents down-regulated genes. Data from 3 biological donors with 1 – 2 replicates from 2 independent experiments (n = 5).

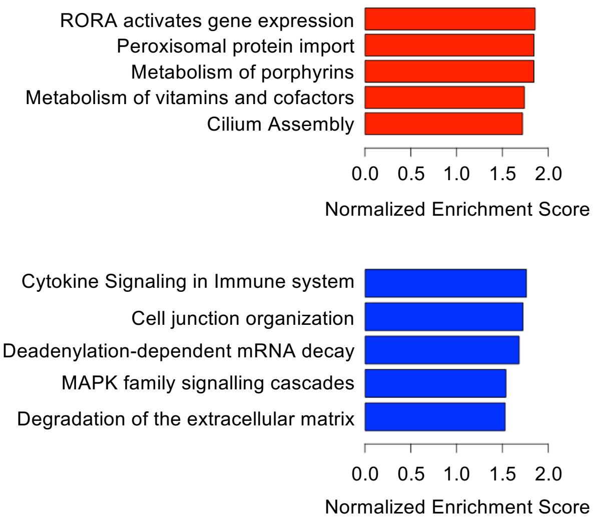


**Figure S2. Genome-wide transcriptome RNAseq analysis of flagellin stimulated HBE cells in the presence of IOX2.** Genome-wide transcriptome analysis of HBE cells stimulated with flagellin for 24 hours in the presence of IOX2 (50 µM) relative to that in the presence of vehicle showing pathways enriched for up-regulated (red) or down-regulated genes (blue). Data from 3 biological donors with 1 – 2 replicates from 2 independent experiments (n = 5).
